# PREDICTORS OF SUICIDE IDEATION AMONG OLDER GHANAIAN WOMEN: RESULTS FROM THE STUDY ON GLOBAL AGEING AND ADULT HEALTH

**DOI:** 10.1093/geroni/igz038.3187

**Published:** 2019-11-08

**Authors:** James Muruthi, Alicia DeLouize, Richard Biritwum, Nirmala naidoo, Paul Kowal, Josh Snodgrass

**Affiliations:** 1 University of Oregon, Eugene, Oregon, United States; 2 University of Oregon, Oregon, United States; 3 University of Ghana, Acrra, Ghana; 4 WHO, Geneva, SD, Switzerland; 5 The University of Newcastle, School of Medicine and Public Health, Faculty of Health and Medicine, Australia

## Abstract

The World Health Organization (WHO) estimates that 79% suicides occur in low- and middle-income countries (LMICs) in 2016 (WHO, 2018) putting a spotlight on the topic of suicide in these countries. While the rates are highest among individuals ages 15 to 29 years, suicide affects individuals, families, and communities throughout the lifespan. The topic of suicide among older adults, especially those living in LMICs, has unfortunately received limited attention. Using a sample of 228 women with depression from the first wave of the WHO’s Study on global AGEing and adult health (SAGE, Ghana), the present study sought to identify predictors of suicide ideation among aging African women. Binominal logistic regression results revealed that wealth (negatively) and health (negatively) were associated with suicide ideation in the sample; indicating that the women who were poor and unhealthy were more likely to have suicidal thoughts. Implications of the findings for aging Ghanaian women will be addressed.

